# Risk management policies and practices regarding radio frequency electromagnetic fields: results from a WHO survey

**DOI:** 10.1093/rpd/ncu324

**Published:** 2014-11-13

**Authors:** Amit Dhungel, Denis Zmirou-Navier, Emilie van Deventer

**Affiliations:** 1 Department of Environmental and Occupational Health, EHESP School of Public Health, Avenue du Professeur Léon Bernard CS 74312, 35043 Rennes, France; 2 Lorraine University School of Medicine, av. de la Forêt de Haye, 54505 Vandoeuvre-Les-Nancy, France; 3 Radiation Programme, Department of Public Health, Environmental and Social Determinants of Health, World Health Organization, Geneva, Switzerland

## Abstract

This study aims to describe current risk management practices and policies across the world in relation to personal exposures from devices emitting radiofrequency fields, environmental exposures from fixed installations and exposures in the work environment. Data from 86 countries representing all WHO regions were collected through a survey. The majority of countries (76.8 %) had set exposure limits for mobile devices, almost all (90.7 %) had set public exposure limits for fixed installations and 76.5 % had specified exposure limits for personnel in occupational settings. A number of other policies had been implemented at the national level, ranging from information provisions on how to reduce personal exposures and restrictions of usage for certain populations, such as children or pregnant women to prevention of access around base stations. This study suggests that countries with higher mobile subscriptions tend to have set radiofrequency exposure limits for mobile devices and to have provisions on exposure measurements about fixed installations.

## INTRODUCTION AND BACKGROUND

There has been ongoing concern about the possibility of adverse health effects resulting from exposure to radiofrequency (RF) electromagnetic fields (EMF), such as those emitted by wireless communication devices and networks in the frequency range of 100 kHz to 300 GHz. There is scientific evidence of demonstrable acute effects (heating) from high levels of exposure, and many countries have developed safety policies based on the exposure limits proposed by the International Committee for Non-Ionizing Radiation Protection (ICNIRP) or Institute of Electrical and Electronics Engineers (IEEE) to prevent such effects^(1–3)^. There is less scientific clarity about the risks of long-term exposure to lower levels of RF EMF^(4)^. In 2012, the International Agency for Research on Cancer classified RF EMF as ‘possibly’ carcinogenic (Group 2B), based on studies on mobile phone usage. To date, there is no international consensus regarding effects of such exposure. In this setting, countries have adopted risk management policies with a variety of national exposure limits reflecting a mix of influences, including scientific evidence, precautionary approaches and local politics.

To assess the status of national policies and regulations on RF EMF and to help policy-makers determine a rational course of action, a survey was undertaken by the Radiation Programme in the Department of Public Health and Environment of the World Health Organization. The survey took place in 2012 in the course of the scientific update of WHO's Environmental Health Criteria (EHC) monograph on RF EMF. The last WHO Monograph on RF EMF was issued in 1993^(5)^, and since that period, there has been a huge propagation in the number of mobile phone users, with an estimated 6.8 billion subscriptions by the end of 2013^(6)^. The survey contributes the WHO's EHC Monograph the basis for health protection against RF EMF, with particular attention to current standards and guidelines, and exhibits the variety of measures in effect worldwide.

This paper summarizes the main findings from this survey and describes key policy actions regarding personal, environmental and occupational exposures to RF fields associated with the following exposure situations:

- Personal exposures associated with the use of mobile devices (such as mobile phones);
- Environmental exposures associated with fixed installations transmitting signals from radio, television and wireless communication networks and
- Occupational exposures in the telecommunication, industrial and medical sectors.

## STUDY METHODS

### Data collection and analysis

An electronic email invitation, with a web link to the on-line survey, was sent through all WHO regional offices to relevant governmental bodies (e.g. Ministry of Health, Ministry of Environment, Ministry of Telecommunications, Ministry of Labor and Radiation Protection Agency). The survey was also sent to the members of the International Advisory Committee of the WHO EMF Project. The comprehensive survey asked over 60 questions related to 3 types of RF exposure categories in 4 of the 6 UN languages (English, French, Spanish and Russian). Information was collected during the period of August to October 2012. A total of 103 responses from 75 countries representing all 6 WHO regions were received. Duplicate responses from individual countries were lumped together. Requests for additional information or clarifications were sent to 45 countries, out of which 33 responded.

A secondary search was carried out, consisting of a review of government policy documents and published literature on radiofrequency policies. The purpose of this secondary search was twofold: (1) to complement missing data from completed WHO surveys and (2) to extract key information from countries that did not participate in the survey. This secondary search focused on countries according to their population size and mobile penetration rate and aimed to ensure geographical balance in terms of participation from all WHO regions. The search was done via Google using keywords. The search retrieved information from 11 additional countries.

With the primary and secondary participation, the total number of respondents was 86. As different countries missed providing responses from one or more questions, the total number of responses for each survey question was different. In this context, the results have been compiled and analysed on a per question basis. The number of respondents for each question is mentioned in the description part of results section.

The data analysis is primarily descriptive. To assess the influence of the penetration of mobile phone usage on national risk management policy, regression analysis was carried out between the proportions of mobile phone subscriptions per 100 inhabitants in each country (source: ITU, 2011)^(7)^ and the prevalence of specific risk management policies. The proportion of mobile phone subscriptions per 100 inhabitants was split into five quintiles: [11.7–87.1], [87.2–106.5], [106.6–117.3], [118.2–131.4] and [132.3–405.4]. The countries in the third quintile and above show high mobile penetration rate, i.e. >100 %, a figure that could be attributed to multiple subscriptions.

## KEY FINDINGS

There were 86 countries included in the survey, of which 10.4 % were from the Africa region, 19.7 % from the Americas, 11.6 % each from Eastern Mediterranean and Western Pacific, 40.7 % from Europe and 6 % from South East Asia. The survey results were presented during an international stakeholders workshop organized in Paris in June 2013, after which all survey respondents were asked during the summer to check for any inaccuracy. Answers were received from 29 countries. Complete results of the survey are posted on the WHO site where all presentations and background documents discussed during the seminar are displayed (www.who.int/peh-emf/meetings/seminar_radiofrequency_june2013/en/).

In terms of study representation, the study covered responses from 44 % (*N* = 86) of the 194 WHO member states that constituted 74.9 % of the global world population. Regarding the regional representativeness, nine countries from the African region responded to the survey representing 83.3 % of the population from the region. The South East Asia region had 11 countries containing approximately a quarter of the world population^(8, 9)^, out of which only 5 countries provided responses to the survey representing 74.8 % of the population of the region. The Western pacific region with 37 countries containing ∼1.8 billion people, i.e. more than one-fourth of the world's population, provided responses from 10 countries representing ∼94 % of the total population in the region. Regarding Europe, which holds ∼12 % of the world population and 53 countries, a total of 35 countries were included, i.e. >90 % of the region population. The representativity is least from the Eastern Mediterranean Region as the responding countries represented only 55.9 % of the region total population.

### Policies on personal exposures from mobile devices

#### Exposure limits for mobile devices

Human exposure limits for mobile devices are measured in terms of specific absorption rate (SAR) in units of Watts per kilogram of body weight. Over two-thirds of responding countries (64.6 %; *N* = 53) reported using ICNIRP international guidelines, including member states of the European Union following the Radio and Telecommunications Terminal Equipment (R&TTE) Directive. Seven countries (Bolivia, Chile, Honduras, India, Republic of Korea, Trinidad and Tobago and the USA) reported following the US Federal Communications Commission (FCC) limits, which is based on exposure limits recommended by IEEE and ICES (International Committee for Electromagnetic Safety). Canada and Russia followed their own evidence-based national limits, like Australia whose limits were closely related to the ICNIRP guidelines.

#### Provisions for information on RF exposure from mobile devices to purchasers

Among 82 responding countries, 76.8 % (*N* = 63) required labelling of mobile devices to inform purchasers on SAR values whereas 23.2 % (*N* = 19; Tanzania, Cuba, Nepal, Nigeria, Denmark, Estonia, St. Lucia, Lao PDR, Bosnia and Herzegovina, Timor Leste, Philippines, San Marino, St. Vincent and the Grenadines, Kiribati, South Africa, Pakistan, Colombia, Botswana and British Virgin Islands) declared no policy on personal exposure from mobile devices. Among the countries with information provisions on RF exposure from mobile devices, a total of 33 mentioned having provisions for advising purchasers by displaying the SAR values in one or more ways: in the device information pamphlet (*N* = 23), on the packaging box (*N* = 6), on the device itself (*N* = 4), on the display shelf (*N* = 2) or on an Internet website (*N* = 14).

#### Provisions for advising the public on how to reduce personal exposure to RF fields emitted by mobile devices

Of 78 countries responding to this question, 45 had provisions for providing advice on limiting RF exposure. The target public was usually the general population, but some countries tailor messages to specific groups, including children (*N* = 23), pregnant women (*N* = 9) and people with biomedical devices (*N* = 14). In most cases, these messages were delivered directly by a ministry or other national authority.

The most common information provided included suggestions to use hands-free kits (69.0 %), to reduce call time (44.4 %), use text messaging (35.6 %), avoid calling with low signals (24.4 %) or use phones with low SAR (22.2 %). This and additional technical information on SAR and RF studies (31.1 %) were provided to the public primarily through printed materials (84.0 %), websites (65.9 %) or mass media (43.2 %).

#### Provisions on information and voluntary limitations of use of mobile phones by children

No country reported a complete ban on mobile phone use by children. However, 36 % of surveyed countries (*N* = 28) encouraged voluntary measures or made other recommendations on the use of mobile phone by children. Among these, 53.6 % (*N* = 15) aimed to moderate the use of mobile phone among children by providing information to parents. Two countries (Russia and Zambia) declared that they have set advisory age limits for usage of mobile phones, whereas in France, a legal provision bans advertisements promoting the sale or use of mobile phones by people under 14.

### Policies on environmental exposures associated with fixed installations transmitting RF signals

#### Exposure limits for fixed installations

Of 86 countries evaluated, almost all (90.7 %; *N* = 78) had set public exposure limits for fixed installations. The majority of countries followed the ICNIRP guidelines (*N* = 57, 66.3 %); five (Armenia, Canada, China, USA and Russia) had set their own standards. Trinidad and Tobago follow the US FCC limits. There were 16 countries reporting that the established exposure limits were lower than the international guidelines, either under an ALARA (‘As Low As Reasonably Achievable’) principle (*N* = 3, 3.5 %) or a precautionary approach (*N* = 11, 12.8 %). Among the countries that declared no exposure limit for fixed installations, all except Syria also had not defined exposure limits for mobile devices.

#### Provisions to prevent public access to areas around fixed installations

Among the 77 countries evaluated, 76.6 % (*N* = 59) mentioned that provisions are in place to prevent public access close to fixed installations. The most common provisions were physical barriers and warning signs (72.9 % for both); some countries also mentioned safety zones and access to accredited personnel only.

#### Requirement for RF measurements around fixed installations

Periodic RF measurements are most often done to ensure that radiation emissions are within the defined exposure limits. Of 79 respondents, 81 % (*N* = 64) mentioned that they have provisions to request RF measurements around fixed installation sites. While some countries, such as the USA, do not have a national provision, some states or cities (e.g. San Francisco) require RF measurements at fixed sites.

Recording of exposure measurements or of modelling results is required in 47 of 78 countries who responded to this question. Records apply to all fixed installations in 19 countries and to mobile phone base stations for the same number of countries. In some nations, this requirement applies when emissions from base stations are over a certain effective isotropic radiated power value. Regulatory provisions allow access to these records for a variety of parties, i.e. a dedicated national agency (*N* = 30) of cases, national or provincial authorities (*N* = 30), local authorities or municipalities (*N* = 23) and the general public (*N* = 19).

#### Procedures prior to installing fixed installations

In 82.5 % of countries (*N* = 66), a specific authorization was required prior to installing a fixed RF emitting installation. In most cases, this authorization scheme concerns all RF installations, but some countries restricted this scheme to mobile phone base stations according to some criteria (e.g. mast height or transmission power) or to broadcasting/radio communication stations alone. While the requirement for authorization applied to all locations in the vast majority of countries where such scheme applies, it could be limited to certain environments such as close to health care facilities (*N* = 4), schools (*N* = 3) or landmarks (*N* = 1).

Half of the responding countries (52 %, *N* = 40) had provisions regarding the spatial distribution of fixed installations. Among these, 76.9 % (*N* = 30) encourage collocation of different operators and 33.3 % (*N* = 13) mention that there can be some restriction regarding the siting of the installations, based on environmental and architectural considerations.

#### Requirements for informing or consulting stakeholders

Among the 75 countries that responded to this question, a little less than half (46.7 %; *N* = 35) mentioned there were requirements for informing or consulting stakeholders, in addition to which 9.3 % (*N* = 7) reported that this is common practice even though there is no policy requirement to do so. The most frequently consulted stakeholders (from 68.3 to 24.4 % of countries, in this order) are the local community or building residents, the adjacent land owners, the local municipal authority (compulsory in some countries) and action groups and associations.

#### Provisions to respond to individuals concerned by RF fields from fixed installations

A total of 55 countries (72.4 %) declared that they have provisions for responding to concerned individuals regarding RF EMF fields emitted from fixed installations. Of those, 61.8 % (*N* = 34) provided individualized consultations and responses, 54.6 % (*N* = 30) general information and factsheets and 34.6 % (*N* = 19) measurements of EMF radiation to show compliance with exposure standards. Three countries declared that they had provisions to undertake research and epidemiological surveys on EMF.

### Policies on occupational exposures in the telecommunication, industrial and medical sectors

#### Occupational exposure limits

Among 81 countries that responded to this question, only 23.5 % (*N* = 19) declared that no exposure limit had been specified for workers exposed to RF fields. Where limits had been set, their rationale and references varied, although the vast majority follow the ICNIRP or IEEE guidelines. Five countries (Armenia, Canada, China, Poland and Russia) report that they do not follow international guidelines but have set their own national limits based on scientific evidence. Two (Luxemburg and Estonia) have adopted lower occupational exposure limits based on a precautionary approach. It is to be noted that, during the period of this survey, EU countries were waiting for the implementation of the EC ‘Directive of the European Parliament and of the Council on the minimum health and safety requirements regarding the exposure of workers to the risks arising from physical agents (EMF)’, now enacted by Directive 2013/35/EU, which is to be transposed into the national legislation of all EU member states by July 2016.

Almost two-third of 77 respondents (63.6 %; *N* = 49) had provisions to limit exposure of personnel during maintenance operations in the telecommunication sector. Likewise, 43.4 % (*N* = 33) had provisions to maintain exposure levels below some standard (i.e. usually in line with exposure limit for general public) in the industry sector, and 62.7 % (*N* = 47) countries had similar provisions in the medical sector. Other reported prevention measures include training on safety protocols in 31.3 % of countries (*N* = 10), risk assessment studies in 21.9 % (*N* = 7) and restricted entry in designated areas in 18.8 % (*N* = 6).

#### Prevention provisions for specific groups in occupational environment

Among the 39 countries with provisions for selected groups of workers like pregnant women, workers with biomedical devices and in some cases workers below 18 y of age, 32 specified the provisions available, among which 50 % (*N* = 16) set restrictive exposure limits, which include setting limits in line with those for the general public. In 46.9 % (*N* = 15) countries, provisions existed to shift exposed jobs for pregnant females and 21.9 % (*N* = 7) declare they have provisions to provide paid sick leave if job shifting is not viable (Belgium, Estonia, Denmark, Germany, Greece, Ireland and Jordan).

### Influence of the penetration rate of mobile phones

The authors assessed whether there is an influence of the penetration of mobile phone usage on national risk management policy. Univariate regression analysis was carried out between the proportions of mobile phone subscriptions per 100 inhabitants in each country (source: ITU, 2011)^(5)^, and the prevalence of specific risk management policies split into five quintiles. The quintiles limits were [11.7–87.1], [87.2–106.5], [106.6–117.3], [118.2–131.4] and [132.3–405.4]. In countries above third quintiles, the penetration rate of mobile phones was >100 %, which accounts to the multiple subscriptions.

Four categories of policy actions showed a statistically significant association (*p* < 0.05) with the proportion of subscribers.

1. Existence of exposure limits for mobile devices and fixed installation emissions: countries with the lowest penetration rate did not generally set exposure limits for mobile devices or fixed installations. All countries with higher penetration rates were similar.
2. Provision of information on RF exposure by mobile devices*:* Only countries with the lowest subscription rates tended not to have provisions to inform purchasers of RF-emitting personal devices how to reduce their exposure.
3. Provisions for specific authorization prior to establishing fixed installations: countries with a greater penetration rate tend more frequently to have set authorization provisions prior to establishing fixed installations as opposed to countries where the subscription rate is low (*N* = 86, *p* < 10^−3^).
4. Provisions to request exposure measurements for RF emitted by fixed installations: countries with greater penetration rates more frequently have such provisions (*N* = 86, <10^−3^).

## DISCUSSION

There has been a vast expansion in the use of mobile communications networks over the past decade, with nearly 7 billion cell phone subscribers in 2014 (ITU). For most people, these networks generate the most frequently encountered source of electromagnetic radiation in the radiofrequency spectrum. Though exposure limits to avoid the acute effects of RF EMF have been developed by international bodies, these are not legally binding except where translated into national regulations. Moreover, the lack of clarity on the safety of long-term exposure at much lower levels has added to public concern over exposure, especially when non-voluntary, as with RF EMF from cellular base stations. WHO member states have stated their need for support to the effective function of their communication networks while protecting their citizens from known or possible health risks. In the absence of a global consensus on exposure limits and safety precautions, countries are adopting or adapting their own RF EMF safety policies.

To the best of the authors' knowledge this is the first formal survey of national regulatory practices related to health protection related to RF EMF generated primarily from wireless networks. A total of 86 countries were included in this study (75 took part in the survey as primary respondents and data could be retrieved from public sources to inform key policy provisions for an additional 11 countries), covering 44 % of the 194 WHO member states and hosting 74.9 % of the global population.

Following further validation and modifications by national authorities, findings were presented in an International stakeholders consultation meeting in Paris in 5 June 2014 where countries were once again provided with an opportunity to amend their response.

There are several notable, though not perhaps surprising findings from this study. One is that the existence of international exposure limits proposed by international bodies such as ICNIRP and IEEE/ICES has been fundamental in helping countries adopt these limits or adapt them into broadly similar national regulations to avoid the known risks of high RF EMF exposure. Another is that several countries, especially larger and wealthier ones, tend to develop their own policies, though usually referencing the same evidence base referenced in the published exposure limits. Hence, harmonization of policies, which can be helpful for saving costs and increase public confidence, is likely to remain incomplete. Political pressure in some countries has resulted in substantial deviations away from science-based limits towards more conservative ‘safety’ restrictions. There is no longitudinal data available to indicate whether this is a growing trend, but this indicates the challenge countries face in developing evidence-based policies.

As suggested by this study, countries with the lowest penetration rates tend to be those where national policies are absent. These also tend to be least-developed nations, where national regulatory capacity is weak across the board. Growth in the number of mobile subscriptions seems to have some positive influence in adoption of policies and practices, but the reason why countries in the same region and similar penetration rates adopt different forms of policies and practices, like exposure values and limitation of use, is unclear. This might relate to more general risk management policy options that, in part, express expectations from the general public and the various bodies that compose it, such as consumers and workers' unions, expectations that vary widely across societies, in line with the level of economic and social development, characteristics of the relationships between policy-makers and citizens and other relevant factors. These factors explain why, although based on the same international body of scientific evidence (with its remaining uncertainties), some policy options might lend more towards precautionary approaches while others will give more initiative to the market players. Also, even when based on the same international guidelines, risk management practices can take a variety of practical forms, in order to be effective and workable in different national legal frameworks, notably in relation to other occupational, health, safety and environment regulations.

Little effort has been undertaken, or at least published, on assessing the effectiveness of risk management RF policies. A consequence is that evidence-based benchmarking for local, national or international policies is limited. It is important that such studies be undertaken and their results be publically available, through institutional websites and, preferably through peer-reviewed publications, to allow experience sharing and dissemination.

## CONCLUSION

Several drivers shape risk management policies in a certain social, economic and political context. For this reason, there is always more than one-way to handle an environmental health issue such as exposure to radiofrequency EMF. Further, when devising policy options aimed at reducing or preventing known or suspected risks, regulators and policy-makers also consider the opportunities offered by the development of RF technologies because risk management decisions are always tradeoffs between benefits and costs of different types. Among these costs, attention should be given to unjustified impediments in the development of RF communication technologies due to restrictions that would go beyond compliance with well-grounded health and safety requirements. Such undue restrictions would have important consequences because these technologies contribute heavily to health, economic and social development in all parts of the world, especially in less-industrialized countries and in remote areas.

## ETHICAL CONSIDERATIONS

This study involved the analysis of data collected by WHO, by its direct link with the relevant government officials in the member states; therefore, this research did not collect sensitive information on individuals but rather a national-level health policy, which were published and often freely downloadable from Internet as well. Meanwhile, all respondents were pre-informed that the results will be published on WHO website. The primary author undertook this study as part of his thesis for European Public Health Master's Degree (www.europubhealth.org) jointly from University of Sheffield, UK and EHESP School of Public Health, France with full scholarship from European Commission's Erasmus Mundus programme (Category A Grants).

## FUNDING

This work was supported by the EHESP School of Public Health, the French National Agency for Health Security [research and development grant 2013-CRD-04] and the World Health Organization as a part of the preparation on WHO EHC Monograph on RF EMF.
